# Human Vδ1^+^ T Cells in the Immune Response to *Plasmodium falciparum* Infection

**DOI:** 10.3389/fimmu.2019.00259

**Published:** 2019-02-14

**Authors:** Lars Hviid, Cecilia Smith-Togobo, Benjamin E. Willcox

**Affiliations:** ^1^Centre for Medical Parasitology, Department of Immunology and Microbiology, Faculty of Health and Medical Sciences, University of Copenhagen, Copenhagen, Denmark; ^2^Centre for Medical Parasitology, Department of Infectious Diseases, Rigshospitalet, Copenhagen, Denmark; ^3^Department of Biochemistry, Cell and Molecular Biology, University of Ghana, Legon, Ghana; ^4^Cancer Immunology and Immunotherapy Centre, Institute of Immunology and Immunotherapy, University of Birmingham, Birmingham, United Kingdom

**Keywords:** gamma-delta (γ/δ) T lymphocytes, Vdelta1 gamma delta T cells, malaria, *Plasmodium falciparum*, innate immunity, acquired immunity, immune regulation

## Abstract

Naturally acquired protective immunity to *Plasmodium falciparum* malaria is mainly antibody-mediated. However, other cells of the innate and adaptive immune system also play important roles. These include so-called unconventional T cells, which express a γδ T-cell receptor (TCR) rather than the αβ TCR expressed by the majority of T cells—the conventional T cells. The γδ T-cell compartment can be divided into distinct subsets. One expresses a TCR involving Vγ9 and Vδ2, while another major subset uses instead a TCR composed of Vδ1 paired with one of several types of γ chains. The former of these subsets uses a largely semi-invariant TCR repertoire and responds in an innate-like fashion to pyrophosphate antigens generated by various stressed host cells and infectious pathogens, including *P. falciparum*. In this short review, we focus instead on the Vδ1 subset, which appears to have a more adaptive immunobiology, but which has been much less studied in general and in malaria in particular. We discuss the evidence that Vδ1^+^ cells do indeed play a role in malaria and speculate on the function and specificity of this cell type, which is increasingly attracting the attention of immunologists.

## Introduction

The most serious form of malaria is caused by the hemoprotozoan parasite *Plasmodium falciparum*. The disease is a major humanitarian and economic burden on societies affected by it, mainly in sub-Saharan Africa, and it leads to the death of about half a million children every year (1, 2). Immunity to the disease is gradually acquired after years of exposure and many disease episodes, and is mainly mediated by IgG antibodies targeting the asexual blood stages of the infection, which are responsible for all the clinical symptoms and complications (3–5). T cells are nevertheless also of obvious importance in acquisition of immunity, not least to enable B-cell class switching and affinity maturation.

Most circulating T cells express αβ type T-cell receptors (TCR-αβ), but a minority of T cells instead expresses the alternative γδ TCR heterodimer (TCR-γδ). The pivotal role of αβ T cells in immunity to *P. falciparum* malaria is well-established. The αβ T cells function both directly as cytotoxic effector cells against infected hepatocytes, and indirectly as CD4^+^ helper cells for a variety of innate and adaptive immune responses to all stages of the parasite life cycle in the human host. Much less is known about the function and significance of γδ T cells in this immunity.

The αβ and γδ T-cell compartments share several features. In both, the TCR constitutes the antigen recognition element of the multi-molecular TCR complex, which also includes several signal transduction components, such as CD3. TCR diversity is generated by somatic recombination events during T-cell maturation in the thymus. As for αβ T cells, the TCRs of γδ T cells are clonally distributed, such that each T-cell clone expresses a single, rearranged TCR variant, which determines the antigen specificity of the clone—at least in the case of αβ T cells.

The two compartments also exhibit important differences. Thus, αβ T cells respond predominantly to protein antigens that are processed by antigen-presenting cells (APCs) and subsequently displayed as short peptides bound to major histocompatibility complex (MHC) molecules on the APC surface. In contrast to αβ T cells, which typically express either CD4 or CD8, γδ T cells often express neither, in particular in the Vγ9^+^Vδ2^+^ subset. In keeping with this lack of MHC restriction elements, recognition of antigen by “double-negative” γδ T cells is not MHC-restricted. Furthermore, Vγ9^+^Vδ2^+^ T cells universally respond to non-peptide prenyl pyrophosphate metabolites (termed phospho-antigens, or P-Ag) (6). These antigens, which are produced by a variety of stressed cells (isopentenyl pyrophosphate, IPP, produced via the host mevalonate pathway) and by infectious pathogens, including *P. falciparum* [(E)-4-Hydroxy-3-methyl-but-2-enyl pyrophosphate, HMB-PP, produced via the microbial non-mevalonate pathway] are structurally related. Accordingly, the Vγ9 chains expressed by these cells are relatively invariant (7, 8) due to convergent and recurrent recombinations (9). In addition, the Vγ9^+^Vδ2^+^ TCR repertoire is already restricted from birth, and contains a high proportion of Vγ9 clonotypes that are shared by many clones in a given individual, and conserved between many individuals (i.e., “public” repertoires). Furthermore, the repertoire of these cells does not exhibit dramatic clonotypic focusing in adults relative to neonates (9, 10). The Vγ9^+^Vδ2^+^ T-cell subset, which is usually the dominant γδ T-cell subset in the peripheral blood of healthy individuals without exposure to *P. falciparum*, can thus be described as an “innate-like” T-cell subset.

To date, the Vγ9^+^Vδ2^+^ cells are the γδ T cells that have attracted by far the most attention in relation to malaria (11, 12). However, we focus here instead on a largely complementary subset that is characterized by a TCR composed of Vδ1 paired with a variety of γ-chains, and that appears to adopt a distinct immunobiology relative to the innate-like Vγ9^+^Vδ2^+^ subset (13). Unlike Vγ9^+^Vδ2^+^ T cells, Vδ1^+^ T cells typically constitute a minority (≤20%) of adult peripheral blood γδ T cells. However, the subset is enriched relative to the Vγ9^+^Vδ2^+^ T-cell subset in tissues, where they have been reported to recognize a variety of host and microbial antigens (14–16). Also in marked contrast, the TCR repertoire of Vδ1^+^ T cells—and of Vγ9^neg^Vδ2^+^ T cells (17)—is highly diverse at birth, and largely non-overlapping between individuals (i.e., “private” repertoires). Furthermore, the TCR repertoire of this γδ T-cell subset becomes increasingly focused over time as a result of selective expansion of specific clonotypes, most likely following antigenic stimulation (9, 18–20). The Vδ1 subset therefore appears to be much more “adaptive-like” than the Vγ9^+^Vδ2^+^ subset (21), and it bears substantial similarities to conventional αβ T cells. Nevertheless, there is certainly evidence that Vδ1^+^ T cells play a distinct role from αβ T cells in the immune response to several infections—including *P. falciparum* malaria.

### Increased Proportions and Numbers of Vδ1^+^ T Cells in Malaria Patients and Healthy Residents From Malaria-Endemic Areas

Within a few years of the discovery of the γδ TCR, several groups reported modest but protracted expansions of γδ T cells in adult *P. falciparum* and *P. vivax* patients with little or no previous malaria parasite exposure (22–24). A later study of malarious children from a highly malaria-endemic area and employing a pan-γδ TCR-specific antibody reported similar findings, and did not find significant differences in peripheral blood γδ T-cell frequencies between children with uncomplicated and severe malaria, respectively (25). The authors also reported significantly decreased absolute numbers of γδ T cells at the time of admission to hospital with malaria (regardless of severity), followed by a transient increase to numbers above normal during convalescence. This was also observed among the few adult first-time malaria patients included in the study (25). Overall, the γδ T cell-specific findings appeared similar in patients with or without prior exposure to malaria, and also resembled earlier reports regarding the αβ T-cell response to malaria, namely an inflammation-induced withdrawal of these cells from the peripheral circulation, followed by their release back into the peripheral blood after successful chemotherapy [reviewed in Hviid (26)].

Substantial γδ T-cell subset heterogeneity was also reported (27–30). These early papers indicated that the γδ T-cell response to *P. falciparum* malaria extends beyond Vγ9^+^Vδ2^+^ cells, although that subset remained the dominant one among the non-immune patients that were studied. However, it was reported shortly after that in semi-immune African children and adults with acute *P. falciparum* malaria, the γδ T cells responding *in vivo* are completely dominated by cells expressing Vδ1, with little contribution from Vγ9^+^Vδ2^+^ T cells (31, 32). A study of children and adults from *P. falciparum*-endemic Lao People's Democratic Republic very recently reported similar findings (33). The expanded Vδ1^+^ subset had an activated phenotype, produced pro-inflammatory cytokines, used a diversity of Vγ chains, and showed spectratyping evidence of clonal focusing (31–33). In fact, the Vδ1^+^ subset appeared to dominate even among healthy *P. falciparum*-exposed individuals living in areas with stable transmission of these parasites (20, 34). In the absence of acute malaria, these cells were CD45RA^+^, resting (CD69^neg^ and HLA-DR^neg^), and about half of them were CD8^+^ (in contrast to the majority of Vγ9^+^Vδ2^+^ cells, which are double-negative). They were clonally restricted in most adults, but less so in children (20). They thus appear phenotypically similar to the Vδ1^+^ cells found in epithelia (35). While Vγ9^+^Vδ2^+^ cells from such individuals could respond when stimulated *in vitro* by *P. falciparum* pyrophosphate antigens (34)—similar to Vγ9^+^Vδ2^+^ cells from donors without previous malaria exposure [reviewed in Howard et al. (11)]—this response did not appear very prominent *in vivo*.

### Vδ1^+^ T Cells in Malaria: What Do They See and What Do They Do?

Essentially nothing is known about the function or antigen specificity/specificities of the dominant Vδ1^+^ γδ T-cell subset in *P. falciparum*-exposed individuals (12). A few studies have indicated that these cells might recognize, respond to, and have a direct effector function against infected erythrocytes in a manner resembling Vγ9^+^Vδ2^+^ cells (11, 33, 36). However, already early on May Ho and colleagues speculated that the expansion of Vδ1^+^ T cells in *P. falciparum* malaria might instead involve “unidentified host factors” (29). Their prediction is supported by the findings that Vδ1^+^ cells from parasite-exposed individuals do not respond markedly to *P. falciparum* antigens *in vitro* (34), including the parasite-derived pyrophosphate antigens recognized by Vγ9^+^Vδ2^+^ cells (37, 38).

Although it is not known what drives the expansion and differentiation of the adaptive-like Vδ1^+^ subset in malaria, Vδ1^+^ T-cell expansion has been observed in several other pathological conditions (16). Examples include infections with human immunodeficiency virus (HIV) (39–42), cytomegalovirus (CMV) and other herpes viruses (43–45), *Onchocerca volvulus* parasites (46), as well as autoimmune diseases such as Takayasu arteritis (47), inflammatory bowel disease and Crohn's disease (48, 49). The possibility that the Vδ1^+^ T-cell response in these diseases involves recognition of host-encoded components is supported by studies of CMV. In that infection, Vδ2^neg^ T cells display shared reactivity against both CMV-infected target cells and uninfected epithelial cells, consistent with recognition of host-encoded antigens (50). Moreover, endothelial protein C receptor (EPCR) has been identified as an antigenic target for a Vδ2^neg^ γδ TCR expressed by a clonotype heavily expanded after infection with CMV (51), which is known to infect endothelial tissues. T-cell activation was dependent on integration of TCR/EPCR-mediated signals with a TCR-extrinsic “multi-molecular stress signature” induced upon infection of target cells that included CMV-mediated increases in ICAM-1 and LFA-1 expression. Conceivably, this may represent one route for Vδ2^neg^ γδ T-cell recognition of “stressed self.” It may be of interest in the context of malaria that EPCR has been identified as a clinically important receptor for *P. falciparum*-infected erythrocytes (52, 53).

Dysregulation of the B-cell compartment might constitute another pathogen-induced change that could be sensed by “adaptive-like” γδ T cells. Of relevance, *P. falciparum* malaria, and indeed a number of other diseases associated with Vδ1^+^ T-cell expansions, is characterized by massive B-cell activation, both of B cells that are specific for the infection causing the disease and B cells that are not (54, 55). This often leads to reactivation of latent EBV (and CMV) infection, and further B-cell proliferation (56–58). From this perspective, it is tempting to speculate that the selective expansion of Vδ1^+^ T cells observed in individuals living in areas with stable transmission of *P. falciparum* occurs in response to antigens expressed by activated B cells, perhaps serving as part of an auto-regulatory response to curb excessive B-cell activation and proliferation. In addition, Vδ1^+^ cells can recognize EBV-transformed B-cell lines (59, 60), and EBV infection can result in expansion of clonally restricted Vδ1^+^ cell populations after stem cell transplantation (61, 62). Conceivably, CD1c/TCT.1/Blast-1 might be an antigen recognized by these cells. Thus, CD8^+^ Vδ1^+^ cells heavily expanded *in vitro* have been shown to recognize this antigen (63, 64), which is expressed/upregulated on some activated and transformed B cells (65, 66). This is not least the case in the spleen, where Vδ1^+^ cells are also abundant (67), and further increase in numbers in response to *P. falciparum* malaria (68). In addition, Vδ1^+^ T-cell reactivity to CD1c tetramers has been demonstrated (69), although to date only involving a low percentage of the Vδ1 T-cell repertoire. It therefore remains unclear whether CD1c-specific cells overlap with *in vivo* expanded clonotypes (21). In summary, while other possibilities cannot be discounted, responses to “stressed self” via recognition of host antigens may contribute to Vδ1-mediated adaptive surveillance in the context of malaria, which could be linked to immune, stress-linked, or EBV/CMV-related sequelae of parasite infection. Such adaptive surveillance of stressed self has strong relevance for the proposed role of Vδ1^+^ T cells in cancer (16, 70–72).

## Concluding Remarks

There is an increasing interest in the role of γδ T cells and other similar cells, such as NK cells, in the immune response to malaria (11, 73, 74). However, the Vδ1^+^ subset has attracted only limited attention so far. Based on the ideas and studies highlighted in this review, we believe that there is a strong case for extending the focus of γδ T-cell studies in malaria beyond the innate-like Vγ9^+^Vδ2^+^ subset, to include adaptive-like γδ T cells. Although, we have focused here on Vδ1^+^ T cells, it is worth noting that clonal expansion of γδ T cells that express Vδ2 chains paired with γ-chains other than Vγ9 has been described in a variety of conditions. Those cell populations also appear to display an “adaptive-like” immunobiology, positioning them functionally much closer to Vδ1^+^ cells than to the innate-like Vγ9^+^Vδ2^+^ cell subset [reviewed in Davey et al. (17)]. Moreover, recent data further suggest that γδ T cells that express neither Vδ1 nor Vδ2 (e.g., Vδ3^+^ cells) exhibit features of such adaptive immune subsets (13, 75). In light of this emerging adaptive immunobiological human γδ T-cell paradigm, examining the contributions of γδ T-cell subsets other than Vγ9^+^Vδ2^+^ in the immune response to malaria is an underexplored and important avenue for investigation.

## Author Contributions

All authors listed have made a substantial, direct and intellectual contribution to the work, and approved it for publication.

### Conflict of Interest Statement

The authors declare that the research was conducted in the absence of any commercial or financial relationships that could be construed as a potential conflict of interest.
